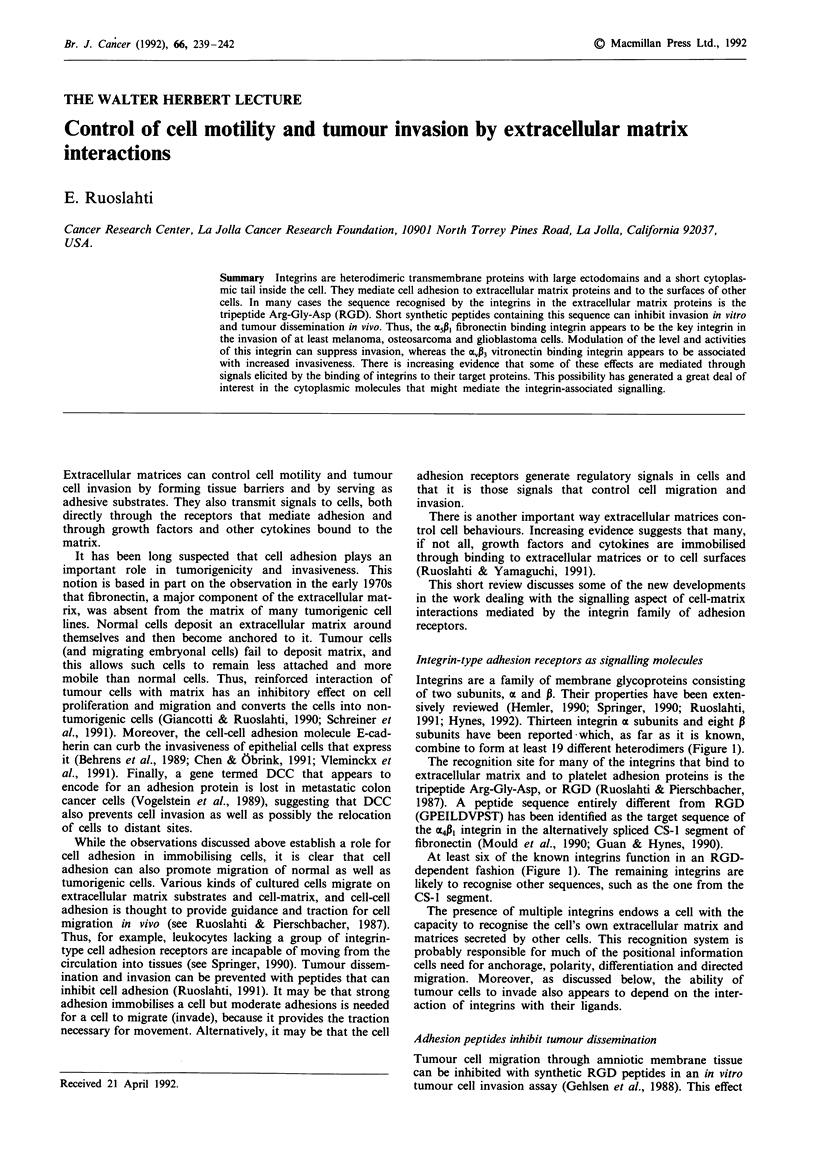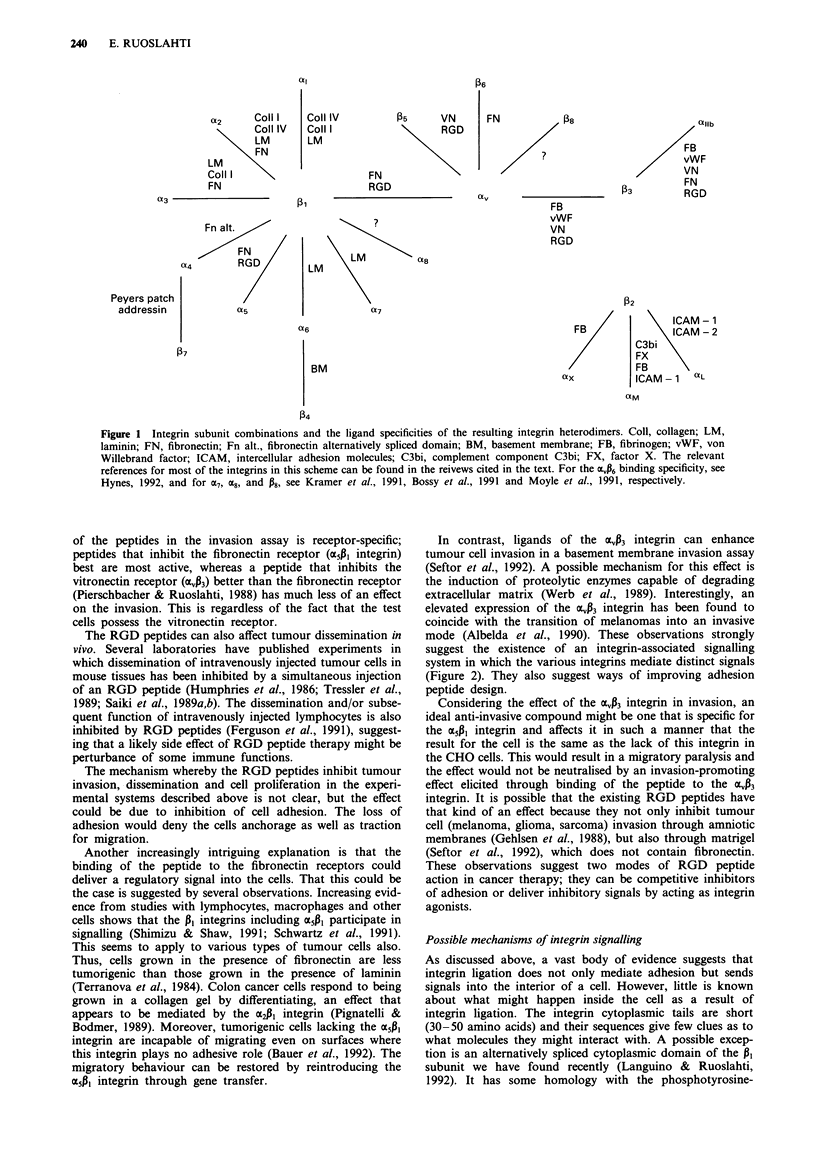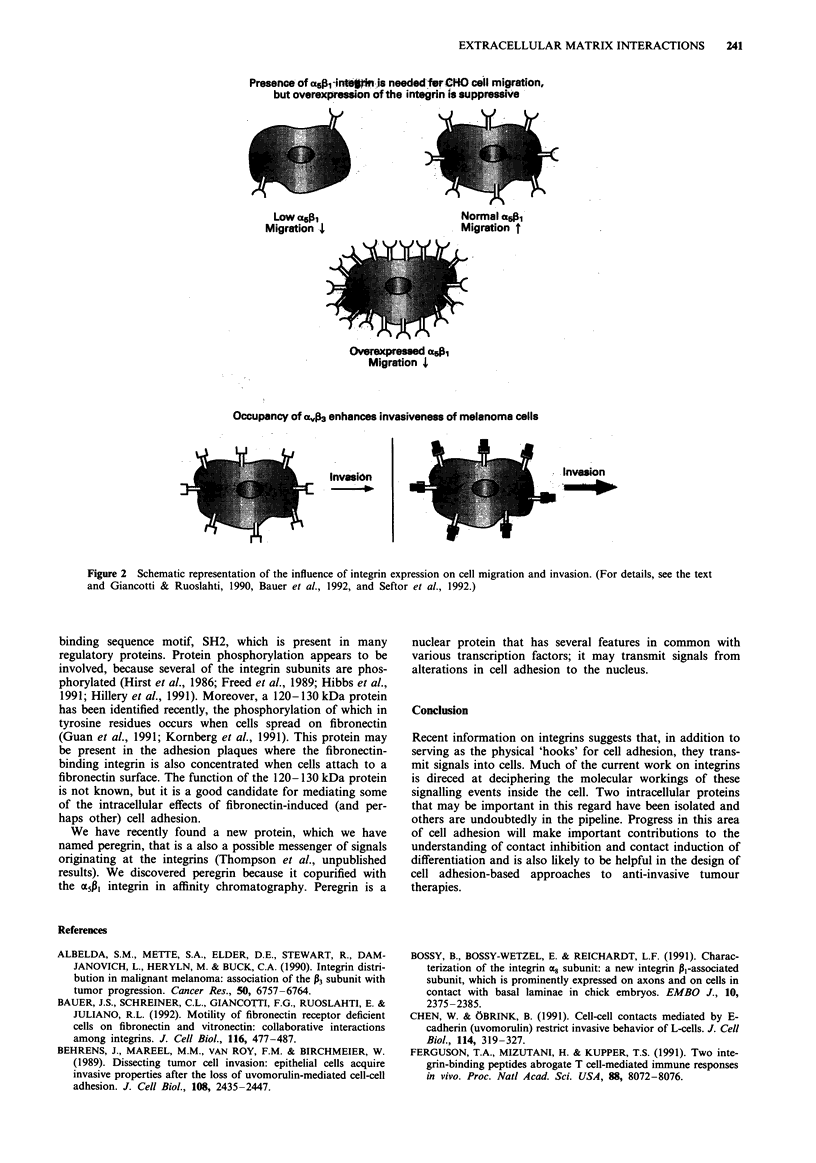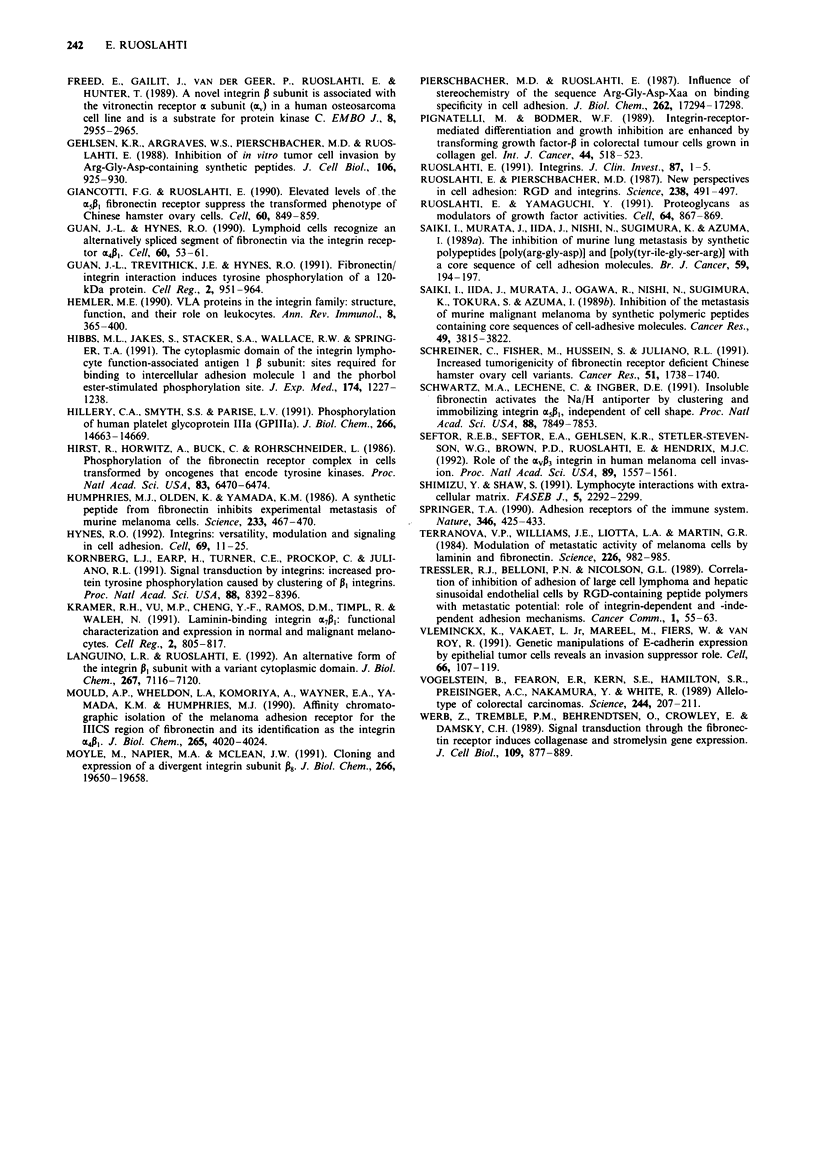# The Walter Herbert Lecture. Control of cell motility and tumour invasion by extracellular matrix interactions.

**DOI:** 10.1038/bjc.1992.250

**Published:** 1992-08

**Authors:** E. Ruoslahti

**Affiliations:** Cancer Research Center, La Jolla Cancer Research Foundation, California 92037.

## Abstract

Integrins are heterodimeric transmembrane proteins with large ectodomains and a short cytoplasmic tail inside the cell. They mediate cell adhesion to extracellular matrix proteins and to the surfaces of other cells. In many cases the sequence recognised by the integrins in the extracellular matrix proteins is the tripeptide Arg-Gly-Asp (RGD). Short synthetic peptides containing this sequence can inhibit invasion in vitro and tumour dissemination in vivo. Thus, the alpha 5 beta 1 fibronectin binding integrin appears to be the key integrin in the invasion of at least melanoma, osteosarcoma and glioblastoma cells. Modulation of the level and activities of this integrin can suppress invasion, whereas the alpha v beta 3 vitronectin binding integrin appears to be associated with increased invasiveness. There is increasing evidence that some of these effects are mediated through signals elicited by the binding of integrins to their target proteins. This possibility has generated a great deal of interest in the cytoplasmic molecules that might mediate the integrin-associated signalling.


 © Macmillan Press Ltd., 1992

THE WALTER HERBERT LECTURE

### Control of cell motility and tumour invasion by extracellular matrix interactions

E. Ruoslahti

*Cancer Research Center, La Jolla Cancer Research Foundation, 10901 North Torrey Pines Road, La Jolla, California 92037, USA.*

**Summary** Integrins are heterodimeric transmembrane proteins with large ectodomains and a short cytoplasmic tail inside the cell. They mediate cell adhesion to extracellular matrix proteins and to the surfaces of other cells. In many cases the sequence recognised by the integrins in the extracellular matrix proteins is the tripeptide Arg-Gly-Asp (RGD). Short synthetic peptides containing this sequence can inhibit invasion *in vitro* and tumour dissemination *in vivo*. Thus, the $\alpha_5\beta_1$ fibronectin binding integrin appears to be the key integrin in the invasion of at least melanoma, osteosarcoma and glioblastoma cells. Modulation of the level and activities of this integrin can suppress invasion, whereas the $\alpha_v\beta_3$ vitronectin binding integrin appears to be associated with increased invasiveness. There is increasing evidence that some of these effects are mediated through signals elicited by the binding of integrins to their target proteins. This possibility has generated a great deal of interest in the cytoplasmic molecules that might mediate the integrin-associated signalling.

Extracellular matrices can control cell motility and tumour cell invasion by forming tissue barriers and by serving as adhesive substrates. They also transmit signals to cells, both directly through the receptors that mediate adhesion and through growth factors and other cytokines bound to the matrix.

It has been long suspected that cell adhesion plays an important role in tumorigenicity and invasiveness. This notion is based in part on the observation in the early 1970s that fibronectin, a major component of the extracellular matrix, was absent from the matrix of many tumorigenic cell lines. Normal cells deposit an extracellular matrix around themselves and then become anchored to it. Tumour cells (and migrating embryonal cells) fail to deposit matrix, and this allows such cells to remain less attached and more mobile than normal cells. Thus, reinforced interaction of tumour cells with matrix has an inhibitory effect on cell proliferation and migration and converts the cells into non-tumorigenic cells (Giancotti & Ruoslahti, 1990; Schreiner *et al.*, 1991). Moreover, the cell-cell adhesion molecule E-cadherin can curb the invasiveness of epithelial cells that express it (Behrens *et al.*, 1989; Chen & Öbrink, 1991; Vleminckx *et al.*, 1991). Finally, a gene termed DCC that appears to encode for an adhesion protein is lost in metastatic colon cancer cells (Vogelstein *et al.*, 1989), suggesting that DCC also prevents cell invasion as well as possibly the relocation of cells to distant sites.

While the observations discussed above establish a role for cell adhesion in immobilising cells, it is clear that cell adhesion can also promote migration of normal as well as tumorigenic cells. Various kinds of cultured cells migrate on extracellular matrix substrates and cell-matrix, and cell-cell adhesion is thought to provide guidance and traction for cell migration *in vivo* (see Ruoslahti & Pierschbacher, 1987). Thus, for example, leukocytes lacking a group of integrin-type cell adhesion receptors are incapable of moving from the circulation into tissues (see Springer, 1990). Tumour dissemination and invasion can be prevented with peptides that can inhibit cell adhesion (Ruoslahti, 1991). It may be that strong adhesion immobilises a cell but moderate adhesions is needed for a cell to migrate (invade), because it provides the traction necessary for movement. Alternatively, it may be that the cell adhesion receptors generate regulatory signals in cells and that it is those signals that control cell migration and invasion.

There is another important way extracellular matrices control cell behaviours. Increasing evidence suggests that many, if not all, growth factors and cytokines are immobilised through binding to extracellular matrices or to cell surfaces (Ruoslahti & Yamaguchi, 1991).

This short review discusses some of the new developments in the work dealing with the signalling aspect of cell-matrix interactions mediated by the integrin family of adhesion receptors.

### *Integrin-type adhesion receptors as signalling molecules*

Integrins are a family of membrane glycoproteins consisting of two subunits, $\alpha$ and $\beta$. Their properties have been extensively reviewed (Hemler, 1990; Springer, 1990; Ruoslahti, 1991; Hynes, 1992). Thirteen integrin $\alpha$ subunits and eight $\beta$ subunits have been reported which, as far as it is known, combine to form at least 19 different heterodimers (Figure 1).

The recognition site for many of the integrins that bind to extracellular matrix and to platelet adhesion proteins is the tripeptide Arg-Gly-Asp, or RGD (Ruoslahti & Pierschbacher, 1987). A peptide sequence entirely different from RGD (GPEILDVPST) has been identified as the target sequence of the $\alpha_4\beta_1$ integrin in the alternatively spliced CS-1 segment of fibronectin (Mould *et al.*, 1990; Guan & Hynes, 1990).

At least six of the known integrins function in an RGD-dependent fashion (Figure 1). The remaining integrins are likely to recognise other sequences, such as the one from the CS-1 segment.

The presence of multiple integrins endows a cell with the capacity to recognise the cell's own extracellular matrix and matrices secreted by other cells. This recognition system is probably responsible for much of the positional information cells need for anchorage, polarity, differentiation and directed migration. Moreover, as discussed below, the ability of tumour cells to invade also appears to depend on the interaction of integrins with their ligands.

### *Adhesion peptides inhibit tumour dissemination*

Tumour cell migration through amniotic membrane tissue can be inhibited with synthetic RGD peptides in an *in vitro* tumour cell invasion assay (Gehlsen *et al.*, 1988). This effect

Received 21 April 1992.

**Figure 1** Integrin subunit combinations and the ligand specificities of the resulting integrin heterodimers. Coll, collagen; LM, laminin; FN, fibronectin; Fn alt., fibronectin alternatively spliced domain; BM, basement membrane; FB, fibrinogen; vWF, von Willebrand factor; ICAM, intercellular adhesion molecules; C3bi, complement component C3bi; FX, factor X. The relevant references for most of the integrins in this scheme can be found in the reivews cited in the text. For the $\alpha_v\beta_6$ binding specificity, see Hynes, 1992, and for $\alpha_7$, $\alpha_8$, and $\beta_8$, see Kramer *et al.*, 1991, Bossy *et al.*, 1991 and Moyle *et al.*, 1991, respectively.

of the peptides in the invasion assay is receptor-specific; peptides that inhibit the fibronectin receptor ($\alpha_5\beta_1$ integrin) best are most active, whereas a peptide that inhibits the vitronectin receptor ($\alpha_v\beta_3$) better than the fibronectin receptor (Pierschbacher & Ruoslahti, 1988) has much less of an effect on the invasion. This is regardless of the fact that the test cells possess the vitronectin receptor.

The RGD peptides can also affect tumour dissemination *in vivo*. Several laboratories have published experiments in which dissemination of intravenously injected tumour cells in mouse tissues has been inhibited by a simultaneous injection of an RGD peptide (Humphries *et al.*, 1986; Tressler *et al.*, 1989; Saiki *et al.*, 1989*a,b*). The dissemination and/or subsequent function of intravenously injected lymphocytes is also inhibited by RGD peptides (Ferguson *et al.*, 1991), suggesting that a likely side effect of RGD peptide therapy might be perturbance of some immune functions.

The mechanism whereby the RGD peptides inhibit tumour invasion, dissemination and cell proliferation in the experimental systems described above is not clear, but the effect could be due to inhibition of cell adhesion. The loss of adhesion would deny the cells anchorage as well as traction for migration.

Another increasingly intriguing explanation is that the binding of the peptide to the fibronectin receptors could deliver a regulatory signal into the cells. That this could be the case is suggested by several observations. Increasing evidence from studies with lymphocytes, macrophages and other cells shows that the $\beta_1$ integrins including $\alpha_5\beta_1$ participate in signalling (Shimizu & Shaw, 1991; Schwartz *et al.*, 1991). This seems to apply to various types of tumour cells also. Thus, cells grown in the presence of fibronectin are less tumorigenic than those grown in the presence of laminin (Terranova *et al.*, 1984). Colon cancer cells respond to being grown in a collagen gel by differentiating, an effect that appears to be mediated by the $\alpha_2\beta_1$ integrin (Pignatelli & Bodmer, 1989). Moreover, tumorigenic cells lacking the $\alpha_5\beta_1$ integrin are incapable of migrating even on surfaces where this integrin plays no adhesive role (Bauer *et al.*, 1992). The migratory behaviour can be restored by reintroducing the $\alpha_5\beta_1$ integrin through gene transfer.

In contrast, ligands of the $\alpha_v\beta_3$ integrin can enhance tumour cell invasion in a basement membrane invasion assay (Seftor *et al.*, 1992). A possible mechanism for this effect is the induction of proteolytic enzymes capable of degrading extracellular matrix (Werb *et al.*, 1989). Interestingly, an elevated expression of the $\alpha_v\beta_3$ integrin has been found to coincide with the transition of melanomas into an invasive mode (Albelda *et al.*, 1990). These observations strongly suggest the existence of an integrin-associated signalling system in which the various integrins mediate distinct signals (Figure 2). They also suggest ways of improving adhesion peptide design.

Considering the effect of the $\alpha_v\beta_3$ integrin in invasion, an ideal anti-invasive compound might be one that is specific for the $\alpha_5\beta_1$ integrin and affects it in such a manner that the result for the cell is the same as the lack of this integrin in the CHO cells. This would result in a migratory paralysis and the effect would not be neutralised by an invasion-promoting effect elicited through binding of the peptide to the $\alpha_v\beta_3$ integrin. It is possible that the existing RGD peptides have that kind of an effect because they not only inhibit tumour cell (melanoma, glioma, sarcoma) invasion through amniotic membranes (Gehlsen *et al.*, 1988), but also through matrigel (Seftor *et al.*, 1992), which does not contain fibronectin. These observations suggest two modes of RGD peptide action in cancer therapy; they can be competitive inhibitors of adhesion or deliver inhibitory signals by acting as integrin agonists.

*Possible mechanisms of integrin signalling*

As discussed above, a vast body of evidence suggests that integrin ligation does not only mediate adhesion but sends signals into the interior of a cell. However, little is known about what might happen inside the cell as a result of integrin ligation. The integrin cytoplasmic tails are short (30–50 amino acids) and their sequences give few clues as to what molecules they might interact with. A possible exception is an alternatively spliced cytoplasmic domain of the $\beta_1$ subunit we have found recently (Languino & Ruoslahti, 1992). It has some homology with the phosphotyrosine-

**Figure 2** Schematic representation of the influence of integrin expression on cell migration and invasion. (For details, see the text and Giancotti & Ruoslahti, 1990, Bauer *et al.*, 1992, and Seftor *et al.*, 1992.)

binding sequence motif, SH2, which is present in many regulatory proteins. Protein phosphorylation appears to be involved, because several of the integrin subunits are phosphorylated (Hirst *et al.*, 1986; Freed *et al.*, 1989; Hibbs *et al.*, 1991; Hillery *et al.*, 1991). Moreover, a 120–130 kDa protein has been identified recently, the phosphorylation of which in tyrosine residues occurs when cells spread on fibronectin (Guan *et al.*, 1991; Kornberg *et al.*, 1991). This protein may be present in the adhesion plaques where the fibronectin-binding integrin is also concentrated when cells attach to a fibronectin surface. The function of the 120–130 kDa protein is not known, but it is a good candidate for mediating some of the intracellular effects of fibronectin-induced (and perhaps other) cell adhesion.

We have recently found a new protein, which we have named peregrin, that is a also a possible messenger of signals originating at the integrins (Thompson *et al.*, unpublished results). We discovered peregrin because it copurified with the $\alpha_5\beta_1$ integrin in affinity chromatography. Peregrin is a nuclear protein that has several features in common with various transcription factors; it may transmit signals from alterations in cell adhesion to the nucleus.

### Conclusion

Recent information on integrins suggests that, in addition to serving as the physical 'hooks' for cell adhesion, they transmit signals into cells. Much of the current work on integrins is direced at deciphering the molecular workings of these signalling events inside the cell. Two intracellular proteins that may be important in this regard have been isolated and others are undoubtedly in the pipeline. Progress in this area of cell adhesion will make important contributions to the understanding of contact inhibition and contact induction of differentiation and is also likely to be helpful in the design of cell adhesion-based approaches to anti-invasive tumour therapies.

### References

ALBELDA, S.M., METTE, S.A., ELDER, D.E., STEWART, R., DAMJANOVICH, L., HERYLN, M. & BUCK, C.A. (1990). Integrin distribution in malignant melanoma: association of the $\beta_3$ subunit with tumor progression. *Cancer Res.*, **50**, 6757–6764.

BAUER, J.S., SCHREINER, C.L., GIANCOTTI, F.G., RUOSLAHTI, E. & JULIANO, R.L. (1992). Motility of fibronectin receptor deficient cells on fibronectin and vitronectin: collaborative interactions among integrins. *J. Cell Biol.*, **116**, 477–487.

BEHRENS, J., MAREEL, M.M., VAN ROY, F.M. & BIRCHMEIER, W. (1989). Dissecting tumor cell invasion: epithelial cells acquire invasive properties after the loss of uvomorulin-mediated cell-cell adhesion. *J. Cell Biol.*, **108**, 2435–2447.

BOSSY, B., BOSSY-WETZEL, E. & REICHARDT, L.F. (1991). Characterization of the integrin $\alpha_8$ subunit: a new integrin $\beta_1$-associated subunit, which is prominently expressed on axons and on cells in contact with basal laminae in chick embryos. *EMBO J.*, **10**, 2375–2385.

CHEN, W. & ÖBRINK, B. (1991). Cell-cell contacts mediated by E-cadherin (uvomorulin) restrict invasive behavior of L-cells. *J. Cell Biol.*, **114**, 319–327.

FERGUSON, T.A., MIZUTANI, H. & KUPPER, T.S. (1991). Two integrin-binding peptides abrogate T cell-mediated immune responses *in vivo*. *Proc. Natl Acad. Sci. USA*, **88**, 8072–8076.

FREED, E., GAILIT, J., VAN DER GEER, P., RUOSLAHTI, E. & HUNTER, T. (1989). A novel integrin $\beta$ subunit is associated with the vitronectin receptor $\alpha$ subunit ($\alpha_v$) in a human osteosarcoma cell line and is a substrate for protein kinase C. *EMBO J.*, **8**, 2955–2965.

GEHLSEN, K.R., ARGRAVES, W.S., PIERSCHBACHER, M.D. & RUOSLAHTI, E. (1988). Inhibition of *in vitro* tumor cell invasion by Arg-Gly-Asp-containing synthetic peptides. *J. Cell Biol.*, **106**, 925–930.

GIANCOTTI, F.G. & RUOSLAHTI, E. (1990). Elevated levels of the $\alpha_5\beta_1$ fibronectin receptor suppress the transformed phenotype of Chinese hamster ovary cells. *Cell*, **60**, 849–859.

GUAN, J.-L. & HYNES, R.O. (1990). Lymphoid cells recognize an alternatively spliced segment of fibronectin via the integrin receptor $\alpha_4\beta_1$. *Cell*, **60**, 53–61.

GUAN, J.-L., TREVITHICK, J.E. & HYNES, R.O. (1991). Fibronectin/integrin interaction induces tyrosine phosphorylation of a 120-kDa protein. *Cell Reg.*, **2**, 951–964.

HEMLER, M.E. (1990). VLA proteins in the integrin family: structure, function, and their role on leukocytes. *Ann. Rev. Immunol.*, **8**, 365–400.

HIBBS, M.L., JAKES, S., STACKER, S.A., WALLACE, R.W. & SPRINGER, T.A. (1991). The cytoplasmic domain of the integrin lymphocyte function-associated antigen 1 $\beta$ subunit: sites required for binding to intercellular adhesion molecule 1 and the phorbol ester-stimulated phosphorylation site. *J. Exp. Med.*, **174**, 1227–1238.

HILLERY, C.A., SMYTH, S.S. & PARISE, L.V. (1991). Phosphorylation of human platelet glycoprotein IIIa (GPIIIa). *J. Biol. Chem.*, **266**, 14663–14669.

HIRST, R., HORWITZ, A., BUCK, C. & ROHRSCHNEIDER, L. (1986). Phosphorylation of the fibronectin receptor complex in cells transformed by oncogenes that encode tyrosine kinases. *Proc. Natl Acad. Sci. USA*, **83**, 6470–6474.

HUMPHRIES, M.J., OLDEN, K. & YAMADA, K.M. (1986). A synthetic peptide from fibronectin inhibits experimental metastasis of murine melanoma cells. *Science*, **233**, 467–470.

HYNES, R.O. (1992). Integrins: versatility, modulation and signaling in cell adhesion. *Cell*, **69**, 11–25.

KORNBERG, L.J., EARP, H., TURNER, C.E., PROCKOP, C. & JULIANO, R.L. (1991). Signal transduction by integrins: increased protein tyrosine phosphorylation caused by clustering of $\beta_1$ integrins. *Proc. Natl Acad. Sci. USA*, **88**, 8392–8396.

KRAMER, R.H., VU, M.P., CHENG, Y.-F., RAMOS, D.M., TIMPL, R. & WALEH, N. (1991). Laminin-binding integrin $\alpha_7\beta_1$: functional characterization and expression in normal and malignant melanocytes. *Cell Reg.*, **2**, 805–817.

LANGUINO, L.R. & RUOSLAHTI, E. (1992). An alternative form of the integrin $\beta_1$ subunit with a variant cytoplasmic domain. *J. Biol. Chem.*, **267**, 7116–7120.

MOULD, A.P., WHELDON, L.A, KOMORIYA, A., WAYNER, E.A., YAMADA, K.M. & HUMPHRIES, M.J. (1990). Affinity chromatographic isolation of the melanoma adhesion receptor for the IIICS region of fibronectin and its identification as the integrin $\alpha_4\beta_1$. *J. Biol. Chem.*, **265**, 4020–4024.

MOYLE, M., NAPIER, M.A. & MCLEAN, J.W. (1991). Cloning and expression of a divergent integrin subunit $\beta_8$. *J. Biol. Chem.*, **266**, 19650–19658.

PIERSCHBACHER, M.D. & RUOSLAHTI, E. (1987). Influence of stereochemistry of the sequence Arg-Gly-Asp-Xaa on binding specificity in cell adhesion. *J. Biol. Chem.*, **262**, 17294–17298.

PIGNATELLI, M. & BODMER, W.F. (1989). Integrin-receptor-mediated differentiation and growth inhibition are enhanced by transforming growth factor-$\beta$ in colorectal tumour cells grown in collagen gel. *Int. J. Cancer*, **44**, 518–523.

RUOSLAHTI, E. (1991). Integrins. *J. Clin. Invest.*, **87**, 1–5.

RUOSLAHTI, E. & PIERSCHBACHER, M.D. (1987). New perspectives in cell adhesion: RGD and integrins. *Science*, **238**, 491–497.

RUOSLAHTI, E. & YAMAGUCHI, Y. (1991). Proteoglycans as modulators of growth factor activities. *Cell*, **64**, 867–869.

SAIKI, I., MURATA, J., IIDA, J., NISHI, N., SUGIMURA, K. & AZUMA, I. (1989*a*). The inhibition of murine lung metastasis by synthetic polypeptides [poly(arg-gly-asp)] and [poly(tyr-ile-gly-ser-arg)] with a core sequence of cell adhesion molecules. *Br. J. Cancer*, **59**, 194–197.

SAIKI, I., IIDA, J., MURATA, J., OGAWA, R., NISHI, N., SUGIMURA, K., TOKURA, S. & AZUMA, I. (1989*b*). Inhibition of the metastasis of murine malignant melanoma by synthetic polymeric peptides containing core sequences of cell-adhesive molecules. *Cancer Res.*, **49**, 3815–3822.

SCHREINER, C., FISHER, M., HUSSEIN, S. & JULIANO, R.L. (1991). Increased tumorigenicity of fibronectin receptor deficient Chinese hamster ovary cell variants. *Cancer Res.*, **51**, 1738–1740.

SCHWARTZ, M.A., LECHENE, C. & INGBER, D.E. (1991). Insoluble fibronectin activates the Na/H antiporter by clustering and immobilizing integrin $\alpha_5\beta_1$, independent of cell shape. *Proc. Natl Acad. Sci. USA*, **88**, 7849–7853.

SEFTOR, R.E.B., SEFTOR, E.A., GEHLSEN, K.R., STETLER-STEVENSON, W.G., BROWN, P.D., RUOSLAHTI, E. & HENDRIX, M.J.C. (1992). Role of the $\alpha_V\beta_3$ integrin in human melanoma cell invasion. *Proc. Natl Acad. Sci. USA*, **89**, 1557–1561.

SHIMIZU, Y. & SHAW, S. (1991). Lymphocyte interactions with extracellular matrix. *FASEB J.*, **5**, 2292–2299.

SPRINGER, T.A. (1990). Adhesion receptors of the immune system. *Nature*, **346**, 425–433.

TERRANOVA, V.P., WILLIAMS, J.E., LIOTTA, L.A. & MARTIN, G.R. (1984). Modulation of metastatic activity of melanoma cells by laminin and fibronectin. *Science*, **226**, 982–985.

TRESSLER, R.J., BELLONI, P.N. & NICOLSON, G.L. (1989). Correlation of inhibition of adhesion of large cell lymphoma and hepatic sinusoidal endothelial cells by RGD-containing peptide polymers with metastatic potential: role of integrin-dependent and -independent adhesion mechanisms. *Cancer Comm.*, **1**, 55–63.

VLEMINCKX, K., VAKAET, L. Jr, MAREEL, M., FIERS, W. & VAN ROY, R. (1991). Genetic manipulations of E-cadherin expression by epithelial tumor cells reveals an invasion suppressor role. *Cell*, **66**, 107–119.

VOGELSTEIN, B., FEARON, E.R, KERN, S.E., HAMILTON, S.R., PREISINGER, A.C., NAKAMURA, Y. & WHITE, R. (1989) Allelotype of colorectal carcinomas. *Science*, **244**, 207–211.

WERB, Z., TREMBLE, P.M., BEHRENDTSEN, O., CROWLEY, E. & DAMSKY, C.H. (1989). Signal transduction through the fibronectin receptor induces collagenase and stromelysin gene expression. *J. Cell Biol.*, **109**, 877–889.